# Retraction: Evaluation of post-COVID mortality risk in cases classified as severe acute respiratory syndrome in Brazil: a longitudinal study for medium and long term

**DOI:** 10.3389/fmed.2025.1619451

**Published:** 2025-05-16

**Authors:** Frontiers Editorial Office

The journal retracts the 18 December 2024 article cited above.

Following publication, concerns were raised regarding the scientific validity of the article. An investigation was conducted in accordance with Frontiers' policies.

The authors failed to provide a satisfactory explanation and as a result, the conclusions of the article have been deemed unreliable and the article has been retracted.

Furthermore, Luiz Antônio Camacho requested to be removed from the Acknowledgements section stating that, though consulted on an early draft of the manuscript, did not endorse the methods in the study.

The Postgraduate Program in Public Health of the Escola Nacional de Saúde Pública Sergio Arouca da Fundação Oswaldo Cruz (PPSP/ENSP/FIOCRUZ) requested to be removed from the Acknowledgments and Funding sections, stating that the article did not receive funding for research or publication.

This retraction was approved by the Chief Editors of Frontiers in Medicine and the Chief Executive Editor of Frontiers. The authors agree to this retraction.

